# An extensible deep learning framework for multi-type chromatin interaction prediction through hierarchical adaptation strategies

**DOI:** 10.1371/journal.pcbi.1014911

**Published:** 2026-10-27

**Authors:** Shuaibin Wang, Tong Chen, Weixu Liu, Xuejie Zheng, Zhongxin Yang, Zhen Liang, Yin Shen

**Affiliations:** 1 School of Biomedical Engineering, Anhui Medical University, Hefei, Anhui, China; 2 Department of Computer Science and Technology, Anhui Medical University, Hefei, Anhui, China; 3 Department of Pediatrics, The First Affiliated Hospital of Anhui Medical University, Hefei, Anhui, China; 4 The Affiliated Chuzhou Hospital of Anhui Medical University, The First People’s Hospital of Chuzhou, Chuzhou, Anhui, China; 5 Second Affiliated Hospital of Anhui Medical University, Hefei, Anhui, China; Hong Kong University of Science and Technology School of Science, HONG KONG

## Abstract

Three-dimensional chromatin interactions play essential roles in gene regulation, yet computational prediction of diverse interaction types remains largely dependent on specialized, single-task models. Here, we present UniLoopNet, a multi-task deep learning framework that predicts multiple types of chromatin interactions including enhancer–promoter interactions, enhancer–enhancer interactions, CTCF-mediated loops, and stripe-associated interactions from DNA sequences. UniLoopNet employs a shared encoder–decoder architecture with hierarchical adaptation strategies, integrating multi-task, transfer, and continual learning based on task semantic similarity. Comprehensive benchmarking across multiple cell lines demonstrates that UniLoopNet outperforms existing specialized and multi-task approaches, with AUROCs exceeding 0.90 in most tasks. Under stringent evaluation settings, including chromosome-matched, distance-matched, realistic imbalanced, and locus-disjoint tests, AUROCs range from 0.8934 to 0.9366. In the realistic 1:5 class-imbalance setting, the AUPRCs for enhancer–promoter and enhancer–enhancer interaction prediction are 0.7956 and 0.6935, respectively. Cross-cell-line transfer experiments further show that applying UniLoopNet to another cell line requires adaptation by supervised fine-tuning on target-cell-line data, after which performance is comparable to that of within-cell-line training. Notably, the framework exhibits data efficiency in experiments with substantially reduced training data. Interpretability analyses reveal that the learned sequence features are associated with known transcription factor binding sites and uncover differential regulatory effects of disease-associated variants. UniLoopNet provides a versatile and data-efficient framework for sequence-based prediction and interpretation of diverse 3D genome interactions, complementing existing sequence-to-map approaches while improving the understanding of sequence-encoded regulatory potential.

## Author summary

The three-dimensional organization of the genome plays a critical role in regulating gene expression by bringing distant regulatory regions into physical contact. However, current computational methods for predicting chromatin interactions are usually designed for individual interaction types, such as enhancer–promoter loops or protein-mediated contacts, requiring separate models for each task. This limits their ability to transfer knowledge across related genomic problems, especially when experimental data are limited. Here, we develop UniLoopNet, an extensible deep learning framework that can predict multiple types of chromatin interactions from DNA sequences. Instead of relying on a single learning strategy, UniLoopNet automatically adapts its knowledge-transfer strategy according to the similarity between different prediction tasks, enabling efficient learning of new interaction types. UniLoopNet achieves accurate predictions across diverse chromatin interaction tasks, is data-efficient, and identifies sequence patterns associated with different regulatory interactions. Applying UniLoopNet to disease-associated genetic variants further suggests how non-coding mutations may influence genome organization. Our study thus provides a flexible computational framework for studying genome regulation and interpreting functional genomic variation.

## Introduction

Three-dimensional genome organization is fundamental to gene regulation, enabling precise spatiotemporal control of transcriptional programs [1,2]. Through chromosome conformation capture technologies, particularly Hi-C, researchers have revealed that the genome is hierarchically organized through various chromatin interactions across multiple scales [3]. These interactions, including enhancer-promoter loops, enhancer-enhancer contacts, CTCF/cohesin-mediated loops, and stripe structures, constitute the structural basis for cell-type-specific gene expression and cellular identity [4–10]. Understanding how these diverse interaction types coordinate to establish and maintain gene regulatory networks remains a central challenge in genomics.

While experimental methods such as Hi-C, ChIA-PET, and HiChIP have revolutionized our understanding of genome architecture [11–13], their application at scale remains constrained by substantial technical and economic barriers [14]. The high cost, complex protocols, and limited throughput of these approaches restrict comprehensive analysis across diverse cell types, developmental stages, and disease conditions. This experimental bottleneck has created an urgent need for computational methods that can accurately predict chromatin interactions from DNA sequence.

Deep learning has emerged as a powerful approach for predicting three-dimensional genome features from sequence [15]. Current methods have achieved notable success but remain largely specialized: models target either specific regulatory elements (Enhancer-MDLF, Bert-Promoter) [16,17] or particular interaction types (ChINN and CLNN-Loop for CTCF loops [18,19], EnContact for enhancer-enhancer [20], EPI-Trans for enhancer-promoter [21]). This specialization, while effective for individual tasks, fails to leverage the fundamental interconnectedness of chromatin interactions, which share common cis-regulatory logic, protein-binding patterns, and biophysical constraints [22,23]. Moreover, the prediction of stripe structures, which are dynamic intermediates in loop extrusion that may mediate long-range regulation, remains largely unexplored, with only preliminary methods like Stripenn [7] available and no sequence-based approaches for predicting stripe-associated interactions.

It is important to distinguish two complementary paradigms in computational prediction of 3D genome features. Sequence-to-map models, such as Akita [24], DeepC [25], and Orca [26], predict entire contact frequency matrices from extended genomic sequences, capturing distance-dependent decay and the effects of structural variants. In contrast, pair-classification models, including ChINN [18], TargetFinder [27], and SPEID [28], predict whether a specific pair of genomic loci interact, offering higher precision for defined interaction categories but without modeling the full contact landscape. UniLoopNet belongs to the pair-classification paradigm, with its primary innovation being hierarchical cross-task knowledge transfer rather than a new input representation.

The mechanistic relationships among different chromatin interaction types provide a compelling rationale for multi-task modeling. Recent multi-task frameworks have demonstrated that joint learning across related genomic tasks yields more generalizable representations and improved transfer learning [29–32]. At the sequence-to-map level, models such as Akita and DeepC predict entire contact matrices, while at the task-specific level, frameworks such as AlphaGenome, EPInformer, Enformer, and ChromBPNet have shown that shared architectural components can capture common regulatory features while maintaining task-specific precision. However, no existing pair-classification framework addresses multiple major types of chromatin interactions within a single model, particularly the integration of loop and stripe predictions.

Here we present UniLoopNet, an extensible deep learning framework that predicts multiple types of chromatin interactions from DNA sequence. By employing a shared encoder-decoder architecture with multi-task learning, UniLoopNet captures both universal and interaction-specific sequence features. The model incorporates several key innovations: (1) unsupervised pre-training to learn general chromatin grammar before task-specific fine-tuning; (2) systematic assessment of task similarities to guide optimal transfer learning strategies; (3) continual learning approaches that enable efficient adaptation to new interaction types; and (4) data efficiency, achieving competitive accuracy with only 2% of training data. Through joint prediction of multiple chromatin interaction types, UniLoopNet provides new insights into sequence-encoded regulatory potential, advancing our understanding of the regulatory mechanisms underlying human health and disease.

## Materials and methods

### Ethics statement

This research is based entirely on publicly available datasets. No experiments involving human subjects or animals were conducted. All data processing and analyses were performed in compliance with relevant guidelines for computational research and data mining. The data sources and computational methods are fully documented in the manuscript.

### Data sources and processing

All datasets utilized in this study were obtained from publicly available repositories. Enhancer-promoter interaction (EPI) data for GM12878 and K562 cell lines were retrieved from the TargetFinder project (https://github.com/shwhalen/targetfinder) [27], comprising 3,801 and 3,952 experimentally validated interactions respectively, with balanced positive and negative samples (1:1 ratio). Enhancer annotations for both cell lines were obtained from the FANTOM5 project (https://fantom.gsc.riken.jp/5/) [33] and integrated with ChIA-PET data from the ChINN project (https://github.com/mjflab/chinn) to identify enhancer-enhancer interactions (EEI). Chromatin loops containing two anchors were systematically analyzed to identify overlapping enhancers; interactions were retained when enhancers completely overlapped with either anchor, with duplicates removed to construct the positive set. Negative samples were generated by randomly pairing the left and right anchor-associated enhancer regions within the same chromosome, which corresponds to a chromosome-matched negative sample construction strategy. Existing positive combinations were explicitly excluded during this process. Finally, under the balanced setting with a 1:1 positive-to-negative ratio, we constructed 6,311 EEI pairs for GM12878 and 15,670 EEI pairs for K562.

Furthermore, we incorporated an additional distance-matched negative sample construction strategy by controlling the genomic distance between paired anchors, ensuring that negative samples exhibited a similar distance distribution to positive samples. This strategy prevents the model from making predictions solely based on genomic distance information. For the realistic imbalanced evaluation, we generated negative samples by integrating chromosome-matched and distance-matched strategies, maintaining consistency in chromosome background and genomic distance distribution while constructing negative sample sets that better reflect the sparsity and class imbalance characteristics of real chromatin interactions. We also introduced an anchor-swap negative sample construction strategy, in which anchors from different positive interaction pairs were exchanged while maintaining comparable genomic distances. This approach generated challenging negative samples with similar genomic contexts but lacking true regulatory relationships, enabling a more rigorous evaluation of whether the model truly learned biologically meaningful chromatin interaction patterns.

Stripe structure coordinates (hg38) were obtained from the Expresso database (https://expresso.sustech.edu.cn/) as detected by Stripenn [7,34] and converted to hg19 using CrossMap with the hg38ToHg19.over.chain file [35], resulting in 8,851 filtered stripe annotations for GM12878. Because stripes are continuous band-like structures in Hi-C contact maps rather than intrinsic pairwise interactions, each annotated stripe was converted into an anchor–anchor paired format compatible with the unified model input: the stripe starting region and its corresponding interacting region along the stripe extension were treated as the two interaction anchors. Positive samples were constructed from the stripe-associated interaction pairs defined by the Expresso annotations, and negative samples were generated by matching the chromosome distribution and sequence background characteristics of the positive samples. Under this formulation, the model performs sequence-based prediction of stripe-associated chromatin interactions rather than direct prediction of complete stripe structures from contact maps. CTCF-mediated interactions were compiled from the ChINN project (https://github.com/mjflab/chinn) for positive samples, with negative samples generated following the FusNet protocol [36], yielding 184,088 balanced interaction pairs for GM12878. Additionally, 664 cis enhancer-gene pairs (GEI) identified through CRISPR/Cas9 perturbation coupled with single-cell RNA sequencing in GM12878 cells [37] were included to evaluate model generalization. Because this data resource provides only experimentally validated positive pairs, we additionally constructed a matched set of non-interacting enhancer–gene negative controls using the chromosome-matched strategy, enabling full classification metrics to be reported for the GEI task (Table B in S5 Table). Enhancer (7,917 samples) and promoter (6,764 samples) datasets for GM12878 were obtained from the Enhancer-MDLF and Bert-Promoter projects respectively, both maintaining 1:1 positive-negative ratios. For all balanced binary classification datasets, the number of positive samples was determined first and an equal number of negative samples was then randomly sampled; when the total number of available samples for a task was odd, an exact 1:1 split is impossible, and we adopted a floor-based strategy (Npos = Nneg = ⌊Ntotal/2⌋) in which the single unmatched sample was excluded rather than assigned to either class. Histone modification ChIP-seq data were obtained from the Roadmap Epigenomics project (http://egg2.wustl.edu/roadmap/data/byFileType/peaks/consolidated/narrowPeak) for additional validation analyses (S4 Table).

### Sequence preprocessing and encoding

To ensure consistent input features across tasks while maintaining computational efficiency, all sequences were standardized to 5,000 bp through center-aligned extension for shorter sequences and truncation for longer ones, ensuring no overlap between augmented sequences derived from different sources. DNA sequences were converted to one-hot encoded matrices (5,000 × 4), where each column represents a nucleotide: A[1,0,0,0], C[0,1,0,0], G[0,0,1,0], T[0,0,0,1], with ambiguous bases encoded as N[0,0,0,0] [38]. All datasets except GEI were partitioned into training, validation, and test sets at the interaction-pair level. Because interaction-pair-level splitting can, in principle, allow identical or overlapping anchor regions to appear in both training and test sets, we additionally performed a stringent enhancer–promoter locus-disjoint evaluation for the core EPI and EEI tasks: test-set interactions were re-filtered based on the genomic loci of their enhancer and promoter anchors so that no anchor present in the test set overlapped any anchor in the training set. Under this filtering, 78 of 701 EEI test interactions were removed (623 retained) and 52 of 422 EPI test interactions were removed (370 retained). The filtering procedure and the associated anchor-overlap statistics are provided in Table A in S5 Table. For each stringent negative-sampling setting (distance-matched, anchor-swap, and realistic imbalanced), the training and test sets were reconstructed according to the corresponding strategy, and the model was retrained with the identical pre-specified architecture, training procedure, and hyperparameters as in the main experiments, without any test-set-driven re-tuning, before evaluation on the corresponding independent test set; the locus-disjoint analysis, by contrast, evaluated the trained model from the main experiments directly on the re-filtered test sets without retraining. The GEI dataset, comprising experimentally validated positive pairs together with the matched negative controls described above, was reserved exclusively for external generalization assessment. Training sets were used for model optimization, validation sets for hyperparameter tuning, and test sets for final performance evaluation on unseen data.

### Model architecture

UniLoopNet employs a dual-channel encoder-decoder architecture with shared-specific task predictors for multi-type chromatin interaction modeling. The framework consists of three main components:

Dual-channel sequence encoder and feature transformation: Each input sequence pair (seq1 and seq2) is processed through symmetric convolutional encoders (SequenceEncoder) [39]. The encoder comprises a three-layer convolutional tower, where each layer consists of 1D convolution, ReLU activation, max pooling, and dropout for hierarchical feature extraction. Layer normalization is applied after the first convolutional layer to stabilize intermediate representations. Initial input channels (n = 4) correspond to one-hot encoded DNA sequences. Subsequently, 1 × 1 convolutions project encoder outputs from channel3 to a fixed embedding dimension (embed_dim), yielding seq1_features and seq2_features respectively.

The encoding process follows:


$$\begin{array}{c} H_{\text{1}}^{\left(i\right)}\;=\;{DO}_{\text{1}}\left({MP}_{\text{1}}\left(\sigma \left({Conv}_{\text{1}}\left(X^{\left(i\right)};\;W_{\text{1}},\;b_{\text{1}}\right)\right)\right)\right) \end{array}$$

$$\begin{array}{c} {\tilde{H}}_{\text{1}}^{\left(i\right)}\;=\;LN\left(H_{\text{1}}^{\left(i\right)}\right) \end{array}$$

$$\begin{array}{c} H_{\text{2}}^{\left(i\right)}\;=\;{DO}_{\text{2}}\left({MP}_{\text{2}}\left(\sigma \left({Conv}_{\text{2}}\left({\tilde{H}}_{\text{1}}^{\left(i\right)};\;W_{\text{2}},\;b_{\text{2}}\right)\right)\right)\right) \end{array}$$

$$\begin{array}{c} H_{\text{3}}^{\left(i\right)}\;=\;{DO}_{\text{3}}\left({MP}_{\text{3}}\left(\sigma \left({Conv}_{\text{3}}\left(H_{\text{2}}^{\left(i\right)};\;W_{\text{3}},\;b_{\text{3}}\right)\right)\right)\right) \end{array}$$

where $W_{k}$ and $b_{k}$ denote convolutional kernels and biases for layer k, σ(⋅) represents ReLU activation, ${MP}_{k}(\cdot )$ denotes max pooling, and ${DO}_{k}(\cdot )$ represents dropout. Feature transformation via 1 × 1 convolution yields:

$Z^{\left(i\right)}\;=\;{Conv}_{\text{1}\times \text{1}}\left(H_{\text{3}}^{\left(i\right)};\;W_{proj}^{\left(i\right)},\;b_{proj}^{\left(i\right)}\right)\;\in \;R^{D\times T}$

where D = embed_dim and T represents downsampled temporal steps.

Dual decoder with reconstruction constraints: Mirror decoders (SequenceDecoder) reconstruct original sequences through multi-stage upsampling and transposed convolutions, outputting four-channel probability distributions via BatchNorm and Sigmoid activation. Reconstruction loss employs mean squared error (MSE) for both sequences as an auxiliary training objective:


$$\begin{array}{c} L_{rec}\;=\;L_{rec}^{\left(\text{1}\right)}\;+\;L_{rec}^{\left(\text{2}\right)}\;,\;L_{rec}^{\left(i\right)}\;=\;{\left\|\hat{X^{\left(i\right)}}\;-\;X^{\left(i\right)}\right\|}_{\text{2}}^{\text{2}} \end{array}$$

Shared-specific dual-task predictor: The predictor module (DualTaskPredictor) employs adaptive global average pooling to aggregate temporal features into fixed-length embeddings, concatenates sequence embeddings, and processes them through shared fully connected layers with dropout. Task-specific heads for EPI and EEI output binary classification scores, capturing universal interaction mechanisms while preserving task-specific distinctions.

### Training strategy

Training employs parallel batch sampling from EPI and EEI dataloaders with cycle-based iteration alignment to mitigate task imbalance. Optimization uses Adam (learning rate = 5 × 10^−5^) with two-phase adaptive scheduling: reconstruction loss-based scheduling during pre-training (ReduceLROnPlateau, factor = 0.5, patience = 10, min_lr = 10^−6^) and validation loss-based scheduling during joint optimization (patience = 20, min_lr = 10^−8^).

### Continual learning implementation

To mitigate catastrophic forgetting when learning new tasks, we implement Elastic Weight Consolidation (EWC) [40,41] with the following loss formulation:


$$\begin{array}{c} L_{total}=L_{clf}+\alpha \cdot L_{recon}+\lambda \cdot L_{EWC} \end{array}$$

where $L_{clf}$ denotes the binary cross-entropy classification loss, $L_{recon}$ denotes the reconstruction loss weighted by α, and $L_{EWC}\;=\;\frac{\text{1}}{\text{2}}\sum_{i}F_{i}{\left(\theta_{i}\;-\;\theta_{i}^{*}\right)}^{\text{2}}$ represents EWC regularization. Here, $\theta_{i}$ denotes current parameters, $\theta_{i}^{*}$ represents optimal parameters from previous tasks, $F_{i}$ approximates the Fisher information matrix indicating parameter importance, and λ controls regularization strength. Unless otherwise stated, α = 1.0 and λ = 5000 were used for all continual learning experiments (see Hyperparameter and Architecture Sensitivity Analysis). Fisher information is computed as: $F_{i}\;=\;E_{x\sim D}\left[{\left(\frac{\partial }{\partial \theta_{i}}\;log\;p\left(y\;|\;x\;;\;\theta \right)\right)}^{\text{2}}\right]$ [42].

Forgetting rate is quantified as:


$$\begin{array}{c} {Forgetting}_{task}\;=\;max\left(\text{0},\;\frac{{AUC}_{old}\;-\;{AUC}_{new}}{{AUC}_{old}}\right) \end{array}$$

where ${AUC}_{old}$ and ${AUC}_{new}$ represent performance before and after new task training respectively.

### Performance evaluation

Model performance was assessed using multiple complementary metrics: area under receiver operating characteristic curve (AUROC), area under precision-recall curve (AUPRC), accuracy, precision, recall, and F1 score. For binary classification tasks:


$$\begin{array}{c} Accuracy\;=\;\frac{TP\;+\;TN}{TP\;+\;TN\;+\;FP\;+\;FN} \end{array}$$

$$\begin{array}{c} Precision\;=\;\frac{TP}{TP\;+\;FP} \end{array}$$

$$\begin{array}{c} Recall\;=\;\frac{TP}{TP\;+\;FN} \end{array}$$

$$\begin{array}{c} F\text{1}\;=\;\text{2}\;\cdot \;\frac{Precision\;\times \;Recall}{Precision\;+\;Recall} \end{array}$$

where TP, TN, FP, and FN represent true positives, true negatives, false positives, and false negatives respectively. AUPR was specifically employed to address class imbalance inherent in chromatin interaction datasets:


$$\begin{array}{c} AUPR\;=\;\int Precision(Recall)\;dRecall \end{array}$$

### Model interpretability analysis

In Silico Mutagenesis (ISM): ISM systematically introduces single nucleotide substitutions across input sequences to quantify positional importance [43]. For each position, all possible nucleotide substitutions are evaluated, with prediction differences from the original sequence indicating functional significance. Positions showing substantial prediction changes upon mutation are identified as critical for model discrimination.

SNP Analysis: Functionally validated systemic lupus erythematosus (SLE) risk variants from genome-wide association studies [44] were analyzed by extracting 1kb flanking regions as anchor sequences. Target gene regions potentially regulated through chromatin loops formed the complementary anchors for interaction prediction.

### Baseline model comparison

To ensure a fair and comprehensive comparison, we compared UniLoopNet with several representative baseline models, including AlphaGenome, EPInformer, ChromBPNet, EPI-Trans, and EnContact. All baseline models were adapted according to the methods and implementation details described in their original publications. For models requiring retraining or fine-tuning, we used the same training, validation, and testing data splits as UniLoopNet to ensure that all models were evaluated under identical data partition conditions. For baseline methods relying on specific input formats or pre-trained models, we strictly followed their original implementation procedures, including the recommended input preprocessing methods and model inference strategies, to maintain consistency with the original design principles of each method. Regarding input information, we ensured that all baseline models were evaluated using the same available biological features as UniLoopNet whenever applicable. Necessary preprocessing steps and format conversions were performed according to the input requirements of each model while maintaining consistency in the underlying biological information. The adaptation and evaluation scripts used for these baseline comparisons are provided in the UniLoopNet GitHub repository (https://github.com/shuaibinw/UniLoopNet).

### Hierarchical transfer strategy based on task similarity

To determine the optimal knowledge transfer strategy among different chromatin interaction prediction tasks, we designed a hierarchical transfer strategy based on task similarity. Since different tasks may have distinct input structures and feature distributions, task similarity was not directly calculated in the original input space. Instead, we quantified task relationships in the high-level feature representation space extracted by the corresponding task-specific feature encoders.

For each task, the input sequences were first processed through the task-specific feature extraction module to obtain task-related feature representations. To eliminate the influence of different feature dimensions and sequence lengths across tasks, adaptive average pooling was applied to transform feature maps into unified-dimensional feature vectors $\text{h}\in R^{D}$. The similarity between two tasks was then measured using cosine similarity:


$$\begin{array}{c} \text{Cosine Similarity}({\text{h}}_{A},{\text{h}}_{B})=\frac{{\text{h}}_{A}\cdot {\text{h}}_{B}}{∥{\text{h}}_{A}{∥}_{\text{2}}∥{\text{h}}_{B}{∥}_{\text{2}}} \end{array}$$

where hA and hB denote the feature representations of the two tasks. This similarity measurement is entirely based on learned feature representations and does not incorporate label information, ensuring an unsupervised evaluation of task relationships and avoiding potential label leakage.

Based on the calculated cosine similarity values between each candidate task and the core-task reference representation (Promoter: 0.879, Enhancer: 0.829, CTCF: 0.789, and Stripe: 0.771), we established operational transfer criteria relative to the core EEI/EPI tasks. Candidate tasks with cosine similarity ≥ 0.82 (Promoter and Enhancer) were assigned to backbone transfer, in which the pre-trained encoder is reused and task-specific components are fine-tuned. Tasks with similarity between 0.77 and 0.82 (CTCF and Stripe) were assigned to continual learning with EWC regularization, which permits gradual adaptation to more divergent feature patterns while preventing catastrophic forgetting. Tasks with similarity below 0.77, none of which were present in the current study, would require largely task-specific training. GEI was handled separately by direct weight transfer without additional training, because its task formulation is identical to that of the core paired-anchor classification tasks and its feature distributions show the highest overlap with the EEI/EPI representations (Fig 3E). These thresholds were derived from the empirical similarity distribution in the task feature space and were not tuned on prediction labels or test-set performance.

Under this scheme, tasks whose representations are more similar to those of the core tasks reuse a larger fraction of the pre-trained parameters, whereas less similar tasks rely more on task-specific adaptation. This hierarchical strategy enables UniLoopNet to exploit shared regulatory patterns among related tasks while limiting negative transfer and preserving task-specific characteristics.

## Results

### UniLoopNet employs hierarchical adaptation strategies for multi-type chromatin interaction prediction

UniLoopNet is a multi-task deep learning framework designed to predict diverse chromatin interactions through integration of multi-task learning, transfer learning, and continual learning strategies (Fig 1A, S1 Table). The architecture comprises three core modules: (1) a dual-channel sequence encoder that processes paired anchor sequences through structurally identical encoders, extracting latent features from one-hot encoded DNA sequences and integrating them via shared fully connected layers to learn task-agnostic representations; (2) a dual decoder with reconstruction constraints that ensures encoded features retain critical sequence information by maximizing recovery of original sequences during pre-training, thereby facilitating robust feature learning; and (3) a shared-specific discriminator that employs task-specific classification heads for different chromatin interaction types while enabling feature sharing at lower layers, achieving integrated multi-task modeling. This modular design enables UniLoopNet to capture both universal and task-specific patterns across diverse chromatin interactions.

**Fig 1:**
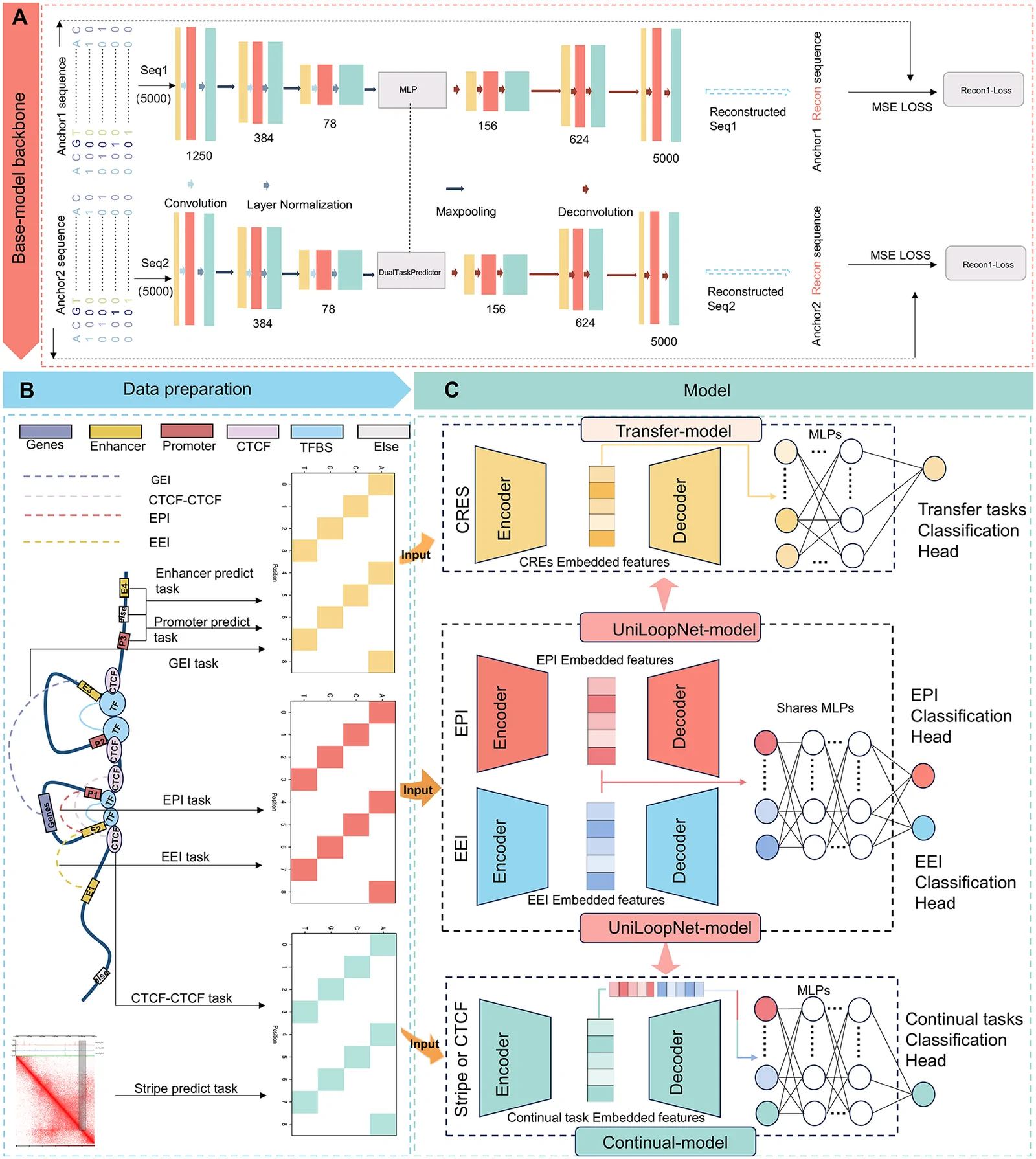
Overview of UniLoopNet architecture and learning strategies. **(A)** UniLoopNet backbone architecture comprising dual symmetric encoders for feature extraction from paired anchor sequences. Parallel autoencoders reconstruct original sequences from extracted features, with mean squared error (MSE) between reconstructed and input sequences providing sequence-level constraints during training. **(B)** Dataset preparation encompassing enhancer-enhancer interactions (EEI), enhancer-promoter interactions (EPI), CTCF-mediated interactions, stripe structures, enhancers, and promoters. All sequences are one-hot encoded to preserve nucleotide-level information before routing to task-specific processing channels. **(C)** Three complementary learning modes: multi-task learning for joint EEI/EPI training (core mode), transfer learning for cis-regulatory element (CRE) adaptation including enhancer and promoter prediction (middle tier), and continual learning for higher-order structural tasks including stripe and CTCF-mediated interactions (foundation tier). These interconnected modes establish an extensible framework for diverse chromatin interaction prediction tasks.

We evaluated UniLoopNet on multiple chromatin interaction datasets including enhancer-enhancer interactions (EEI), enhancer-promoter interactions (EPI), gene-enhancer interactions (GEI), enhancers, promoters, stripes, and CTCF-mediated interactions (Fig 1B). Using EEI and EPI as core training tasks to establish robust multi-task prediction capabilities, we then implemented hierarchical adaptation strategies based on quantitative semantic similarity analysis between tasks (Fig 1C). GEI, whose task formulation matches that of the core paired-anchor tasks and whose feature distributions overlap most strongly with the EEI/EPI representations, employs direct weight transfer from the trained model; enhancer and promoter prediction, which show the highest cosine similarity to the core-task representation among the adaptation tasks (0.829 and 0.879), utilize backbone transfer for knowledge adaptation; and stripe and CTCF-mediated interaction prediction, which show comparatively lower similarity (0.771 and 0.789), apply continual learning to enable gradual adaptation without catastrophic forgetting. This hierarchical framework demonstrates UniLoopNet’s flexibility in cross-task knowledge transfer, with experimental results confirming that all three strategies achieve competitive performance on their respective tasks, validating the framework’s versatility and scalability for diverse chromatin interaction prediction challenges.

### UniLoopNet achieves superior performance in predicting multiple chromatin interaction types across cell lines

We systematically evaluated UniLoopNet’s performance in predicting enhancer-promoter interactions (EPI) and enhancer-enhancer interactions (EEI) across GM12878 and K562 cell lines. Training was conducted for 300 epochs with early stopping to prevent overfitting. UniLoopNet was benchmarked against three representative multi-task methods (AlphaGenome, EPInformer, and ChromBPNet) and two task-specific models (EPI-Trans for EPI prediction and EnContact for EEI prediction). Performance was assessed using area under the receiver operating characteristic curve (AUROC), area under the precision-recall curve (AUPRC), accuracy (ACC), recall, and macro-averaged F1 score (S1 Fig). UniLoopNet outperformed all existing multi-task methods in both cell lines (Fig 2A, 2B). In GM12878, UniLoopNet achieved mean AUROC of 0.9051 and mean AUPRC of 0.9023 for combined EEI and EPI prediction. Performance was even more robust in K562, with mean AUROC and AUPRC reaching 0.9511 and 0.9551, respectively. Notably, UniLoopNet demonstrated competitive performance against task-specific models: it surpassed the EEI-specific EnContact across all five metrics in both cell lines, and while achieving comparable AUROC and AUPRC to the EPI-specific EPI-Trans, showed higher accuracy (Fig 2C). These results highlight UniLoopNet’s capability to accurately identify and distinguish multiple chromatin interaction types while maintaining robust performance in both cell lines.

**Fig 2:**
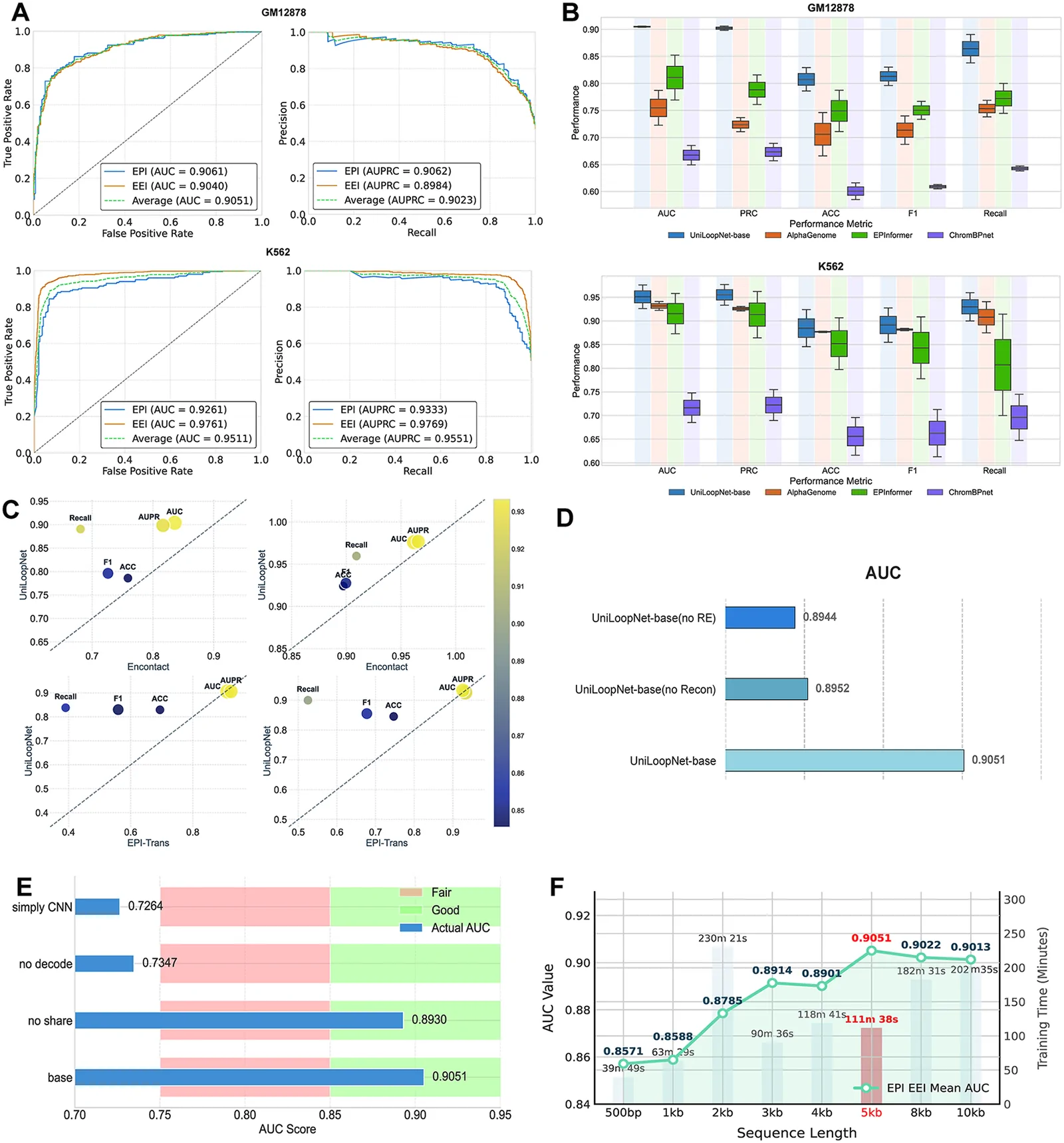
Performance comparison of UniLoopNet with baseline methods. **(A)** AUROC and AUPRC values for EEI and EPI prediction in GM12878 and K562 cell lines. **(B)** Comprehensive performance comparison between UniLoopNet and other multi-task methods across five metrics (AUROC, AUPRC, Accuracy, Recall, F1) in GM12878 and K562 cell lines. Box plots show quartile distributions with median indicated by horizontal line; whiskers extend to 1.5 × interquartile range with outliers marked individually. **(C)** Performance comparison between UniLoopNet and task-specific methods (EnContact for EEI, EPI-Trans for EPI) in GM12878 and K562 cell lines. Bubble chart positions indicate relative performance advantages, with positions closer to y-axis representing superior UniLoopNet performance across more metrics. **(D)** Ablation study of loss functions showing performance changes after removing reconstruction loss or regularization loss. **(E)** Architectural ablation study evaluating impacts of simplified encoder convolutions, decoder removal, and shared layer elimination. **(F)** Mean AUROC across the EPI and EEI tasks (line) and total training time (bars) for input sequence lengths from 500 bp to 10 kb; the 5 kb configuration used throughout this study is highlighted.

To dissect the contributions of key architectural components, we conducted comprehensive ablation studies in GM12878 cells, examining loss functions, model architecture, and input sequence length. Both regularization and reconstruction losses contributed to model performance (Fig 2D): removing regularization loss decreased AUROC from 0.9051 to 0.8944, while removing reconstruction loss reduced it to 0.8952. Architectural ablations revealed that reducing convolutional layers in the backbone network and removing the decoder module severely impacted performance, with AUROC dropping to 0.7264 and 0.7347 respectively, whereas removing the shared layer had minimal effect (Fig 2E).

To determine whether these gains derive from multi-task information sharing rather than from architectural capacity or the reconstruction objective alone, we additionally trained independent single-task models for EPI and EEI with exactly the same network architecture, input data, and training strategy as UniLoopNet, differing only in the removal of cross-task joint optimization. UniLoopNet outperformed the matched single-task models on both tasks (EPI: AUROC 0.9061 versus 0.8898, AUPRC 0.9062 versus 0.8981; EEI: AUROC 0.9040 versus 0.8911, AUPRC 0.8984 versus 0.8854; Table C in S5 Table), indicating that the improvement is attributable to information sharing between related interaction prediction tasks rather than to model capacity or reconstruction regularization alone.

### Performance under stringent negative-sampling and imbalanced evaluation settings

Because balanced test sets with randomly paired negative samples may overestimate predictive performance in realistic scenarios, we further evaluated UniLoopNet in GM12878 under a series of more stringent evaluation settings (S2B Fig). Specifically, for each negative-sampling setting, we reconstructed independent training and test sets according to the corresponding strategy, retrained UniLoopNet using the same model training procedure as in the main experiments, and subsequently evaluated the retrained model on the corresponding independent test set. In addition to the default chromosome-matched negatives, we constructed distance-matched negatives to preserve the genomic distance distribution of positive interaction pairs; we also constructed anchor-swap negatives, in which anchors from different positive interaction pairs were recombined within comparable genomic distance ranges. Furthermore, we constructed a realistic imbalanced test set with a positive-to-negative ratio of 1:5, using a combination of chromosome-matching and distance-matching strategies to generate negative samples (Methods). For the EPI task, UniLoopNet achieved AUROC values of 0.9029, 0.9061, and 0.8980 on the distance-matched, chromosome-matched, and anchor-swap test sets, respectively, with corresponding AUPRC values of 0.9091, 0.9062, and 0.8942. Under the realistic imbalanced setting, the model achieved an AUROC of 0.9366 and an AUPRC of 0.7956. For the EEI task, the corresponding AUROC values were 0.9159, 0.9040, and 0.9036, with AUPRC values of 0.9216, 0.8984, and 0.8904, respectively; under the imbalanced setting, the model achieved an AUROC of 0.9192 and an AUPRC of 0.6935. As expected, AUPRC decreased under class imbalance owing to the reduced proportion of positive samples.

It is important to note that although the training and test sets were reconstructed and the model was retrained separately for each evaluation setting, we did not perform any additional re-tuning or test-set-driven model optimization based on the results obtained from the corresponding test sets. Across all stringent evaluation settings, the model architecture, training strategy, and hyperparameters were kept identical to those pre-specified in the main experiments. Therefore, the results under the different evaluation settings reflect the generalization capability of UniLoopNet under the corresponding data-construction strategies using a fixed, pre-defined modeling scheme, rather than performance gains obtained through test-set-specific optimization. To further exclude the possibility that model performance was attributable to memorization of shared anchors between the training and test sets, we applied the enhancer–promoter locus-disjoint evaluation described in the Methods. In this evaluation, the trained model from the main experiments was evaluated directly, without retraining, on test sets re-filtered at the anchor-locus level. After removing all test interactions whose anchors overlapped with any training anchor (EEI: 701 to 623; EPI: 422 to 370), UniLoopNet retained an AUROC of 0.8967 and an AUPRC of 0.8872 on the filtered EPI test set, and an AUROC of 0.8934 and an AUPRC of 0.8767 on the filtered EEI test set. In comparison, the corresponding values before filtering were 0.9061/0.9062 for EPI and 0.9040/0.8984 for EEI (Table A in S5 Table). This modest decrease indicates that the predictive performance of UniLoopNet is primarily driven by generalizable sequence features rather than by memorization of anchors present in the training data.

### Cross-cell-line adaptation with target-cell-line supervision

To quantify what prediction from DNA sequence can and cannot deliver across cellular contexts, we performed cross-cell-line transfer experiments between GM12878 and K562 (S2C Fig). In all within-cell-line experiments, separate models were trained and tested on data from the same cell line, yielding reference AUROCs of 0.9061 (EPI) and 0.9040 (EEI) in GM12878, and 0.9261 (EPI) and 0.9761 (EEI) in K562. When a model trained solely on K562 was applied directly to GM12878 without any target-cell-line adaptation, performance dropped to AUROCs of 0.6073 (EPI) and 0.8062 (EEI), with an average of 0.7067; after fine-tuning for 15 epochs at a learning rate of 5 × 10^−6^  using the full training set of target-cell-line data, performance recovered to 0.9062 (EPI) and 0.9214 (EEI), with an average of 0.9138, comparable to within-cell-line training. In the reverse direction (GM12878 to K562), direct transfer yielded AUROCs of 0.6233 (EPI) and 0.8889 (EEI), with an average of 0.7561, which improved to 0.9452 (EPI) and 0.9786 (EEI), with an average of 0.9619, after the same fine-tuning procedure. For reference, the EPI-specific model EPI-Trans achieved cross-cell-line AUROCs of 0.616 (K562 to GM12878) and 0.643 (GM12878 to K562) without target adaptation. Together, these results show that DNA sequence alone does not fully capture cell-state-specific chromatin interactions. Applying UniLoopNet to another cell line therefore requires adaptation by supervised fine-tuning on target-cell-line data, after which performance is restored to within-cell-line levels.

### Sequence length optimization and computational efficiency analysis

Furthermore, we systematically evaluated the impact of input sequence length on UniLoopNet performance and computational efficiency (Fig 2F). Since experimentally annotated enhancers, promoters, and other regulatory elements are generally shorter than 5 kb, the 5 kb input window used in this study does not represent the intrinsic length of functional elements, but rather defines the local regulatory genomic environment surrounding chromatin interaction anchors. This window enables the model to capture informative sequence contexts, including nearby transcription factor binding sites, regulatory motifs, and surrounding genomic features associated with chromatin interactions. To validate the rationality of the selected sequence length, we evaluated UniLoopNet using different input sequence lengths (500 bp, 1 kb, 2 kb, 3 kb, 4 kb, 5 kb, 8 kb, and 10 kb) while keeping the model architecture, training strategy, and data partitioning scheme unchanged. The results showed that model performance gradually improved with increasing sequence length and reached the optimal performance at 5 kb, achieving a mean AUC of 0.9051. Further increasing the sequence length to 8 kb and 10 kb did not provide additional improvements, with mean AUC values decreasing to 0.9022 and 0.9013, respectively, suggesting that excessively long sequence windows may introduce redundant genomic information that does not contribute to the prediction task.

To further evaluate the relationship between predictive performance and computational efficiency, we compared the training time required for different sequence lengths under the same computational environment and training strategy. The training times for input lengths of 500 bp, 1 kb, 2 kb, 3 kb, 4 kb, 5 kb, 8 kb, and 10 kb were 39 min 49 s, 63 min 29 s, 230 min 21 s, 90 min 36 s, 118 min 41 s, 111 min 38 s, 182 min 31 s, and 202 min 35 s, respectively. Training time generally increased with input sequence length, although the trend was not strictly monotonic. Notably, the training time for the 2 kb input sequence was substantially higher than those for the neighboring sequence lengths. We further investigated this anomalous result and found that a temporary server-side system latency occurred during the corresponding experiment, resulting in an unusually prolonged training time. Therefore, this anomalous value primarily reflects a measurement deviation caused by the server’s operating condition rather than the actual computational cost associated with the 2 kb sequence length. Considering both predictive performance and computational efficiency, the 5 kb input window achieved the best predictive performance while avoiding the additional computational burden associated with longer sequences, and was therefore selected as the standard input length for UniLoopNet. Overall, these sequence-length ablation experiments and computational efficiency analyses demonstrate that the selection of the 5 kb input window was not based on a predefined assumption, but was determined through systematic evaluation of multiple candidate sequence lengths. These results further support the rationale of the input sequence design and provide experimental justification for the application of UniLoopNet to multi-type chromatin interaction prediction.

### UniLoopNet reveals shared features across chromatin interactions and guides optimal transfer strategy selection

To investigate whether the shared architecture effectively captures common features across different tasks, we analyzed semantic similarities between EEI and EPI anchor sequences before and after encoding. Original sequence similarities showed substantial asymmetry: EEI anchor1 and EPI anchor1 exhibited high similarity (0.745) as both represent enhancers, while EEI anchor2 (enhancer) and EPI anchor2 (promoter) showed lower similarity (0.534) due to their distinct regulatory identities (Fig 3A). Remarkably, after processing through the shared encoder, this asymmetry was substantially reduced, with anchor2 similarity increasing from 0.534 to 0.660, demonstrating that the shared architecture effectively extracts and propagates enhancer features from EEI anchor2, thereby enhancing cross-task feature sharing. The discriminative capacity of UniLoopNet’s task-specific classification heads was validated through t-SNE visualization, revealing clear separation between positive and negative interactions for both tasks (Fig 3B). To assess biological interpretability, we applied UniLoopNet to functionally validated SNPs from systemic lupus erythematosus genome-wide association studies, where massively parallel reporter assays identified variants with genotype-dependent enhancer activity. Differential prediction scores between EEI and EPI heads revealed that certain SNPs preferentially affect specific interaction types (Fig 3C): some variants showed strong EEI signals with weak EPI signals, while others displayed the opposite pattern (S2 Table), suggesting that genetic variants may exert regulatory effects through distinct spatial interaction mechanisms. Notably, rs2069235 (G > A) near the APOBEC3 cluster exhibited an asymmetry: EEI prediction scores decreased across all nine target genes (Δ ranging from −0.0008 to −0.031), whereas EPI effects were more heterogeneous and generally smaller in magnitude (Δ ranging from 0 to −0.0153; S2 Table). This pattern suggests that the variant may selectively weaken enhancer-enhancer regulatory contacts while leaving enhancer-promoter interactions relatively intact, consistent with the hypothesis that certain disease-associated variants exert their effects through specific structural interaction mechanisms rather than globally disrupting chromatin architecture.

**Fig 3:**
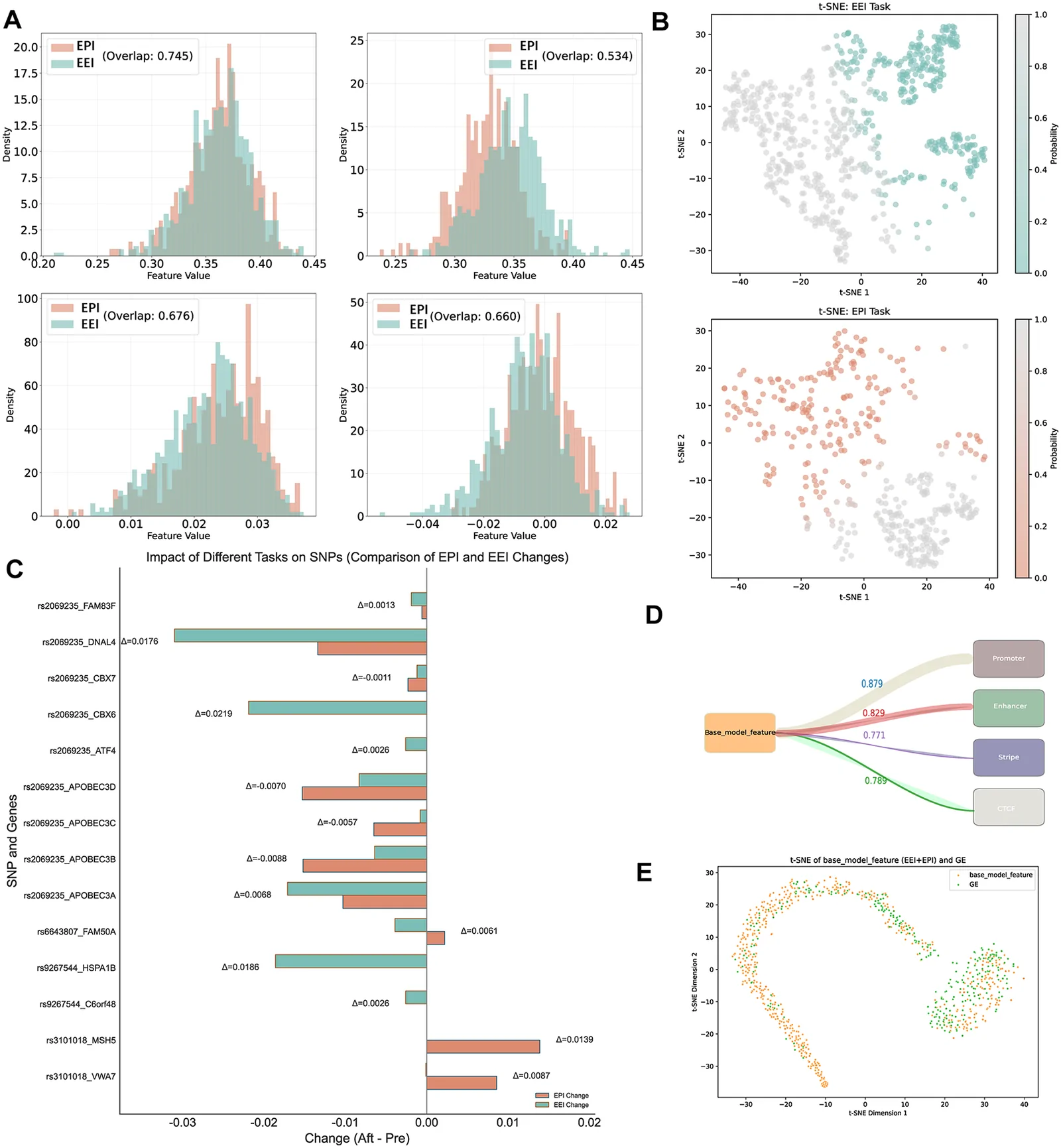
Interpretability analysis of UniLoopNet. **(A)** Semantic feature overlap between EEI and EPI sequences before and after processing through symmetric encoders, demonstrating enhanced feature sharing post-encoding. (B) t-SNE visualization of UniLoopNet outputs for EEI and EPI tasks on test data, showing clear separation between positive and negative interactions. **(C)** Differential regulatory effects of systemic lupus erythematosus (SLE) GWAS risk variants on chromatin interactions. Disease-associated SNPs exhibit distinct influence patterns on EEI versus EPI, suggesting diverse regulatory mechanisms underlying disease susceptibility. **(D)** Semantic similarity analysis between EEI/EPI features and those of enhancers, promoters, stripes, and CTCF binding sites, providing quantitative guidance for transfer strategy selection. **(E)** Feature distribution comparison revealing high similarity between EEI/EPI and gene-enhancer interaction (GEI) patterns.

Systematic analysis of feature distribution similarities and semantic relationships among all tasks provided quantitative guidance for transfer strategy selection (Fig 3D, 3E). Gene-enhancer interactions showed the closest correspondence with the core EEI/EPI representations, enabling direct application of trained UniLoopNet weights without additional training. Enhancer and promoter prediction exhibited the highest cosine similarity to the core-task representation among the single-sequence adaptation tasks (0.829 and 0.879), making them suitable for backbone transfer approaches that leverage pre-trained encoders while fine-tuning task-specific components. In contrast, stripe and CTCF binding site prediction showed comparatively lower similarity scores (0.771 and 0.789), necessitating continual learning strategies to prevent catastrophic forgetting while acquiring task-specific patterns. This similarity-based framework for strategy selection significantly enhanced UniLoopNet’s adaptability across diverse chromatin interaction prediction tasks, with experimental validation confirming that each strategy achieves optimal performance for its designated task category. The hierarchical transfer approach not only improves computational efficiency but also demonstrates how understanding task relationships can guide architectural decisions in multi-task genomic modeling.

### UniLoopNet demonstrates transferability across genomically related tasks

To evaluate UniLoopNet’s generalization capability and architectural versatility, we implemented task-specific adaptation strategies based on feature distribution similarities between tasks (Fig 3E). For gene-enhancer interaction (GEI) prediction, which showed high feature similarity with EEI/EPI, we employed direct weight transfer without additional training (Fig 4A). Evaluation on 664 functionally validated cis enhancer-gene pairs yielded a recall of 0.9187 (S3 Table; Fig 4B). Recall showed minimal fluctuation across different prediction thresholds (0.5–0.9). Because recall on positive-only data cannot by itself establish discriminative performance, we further evaluated UniLoopNet on the GEI task using the matched negative control set described in Methods. On this combined positive–negative test set, the model achieved an AUROC of 0.8922 and an AUPRC of 0.8957, with a recall of 0.8182, a specificity of 0.8182, and a false positive rate of 0.1818 (Table B in S5 Table). Analysis of interaction distances revealed optimal performance at 10kb separation between gene and enhancer. We note that this distance-dependent profile was obtained with the standard 5 kb anchor input window and therefore characterizes the model configuration used throughout this study.

**Fig 4:**
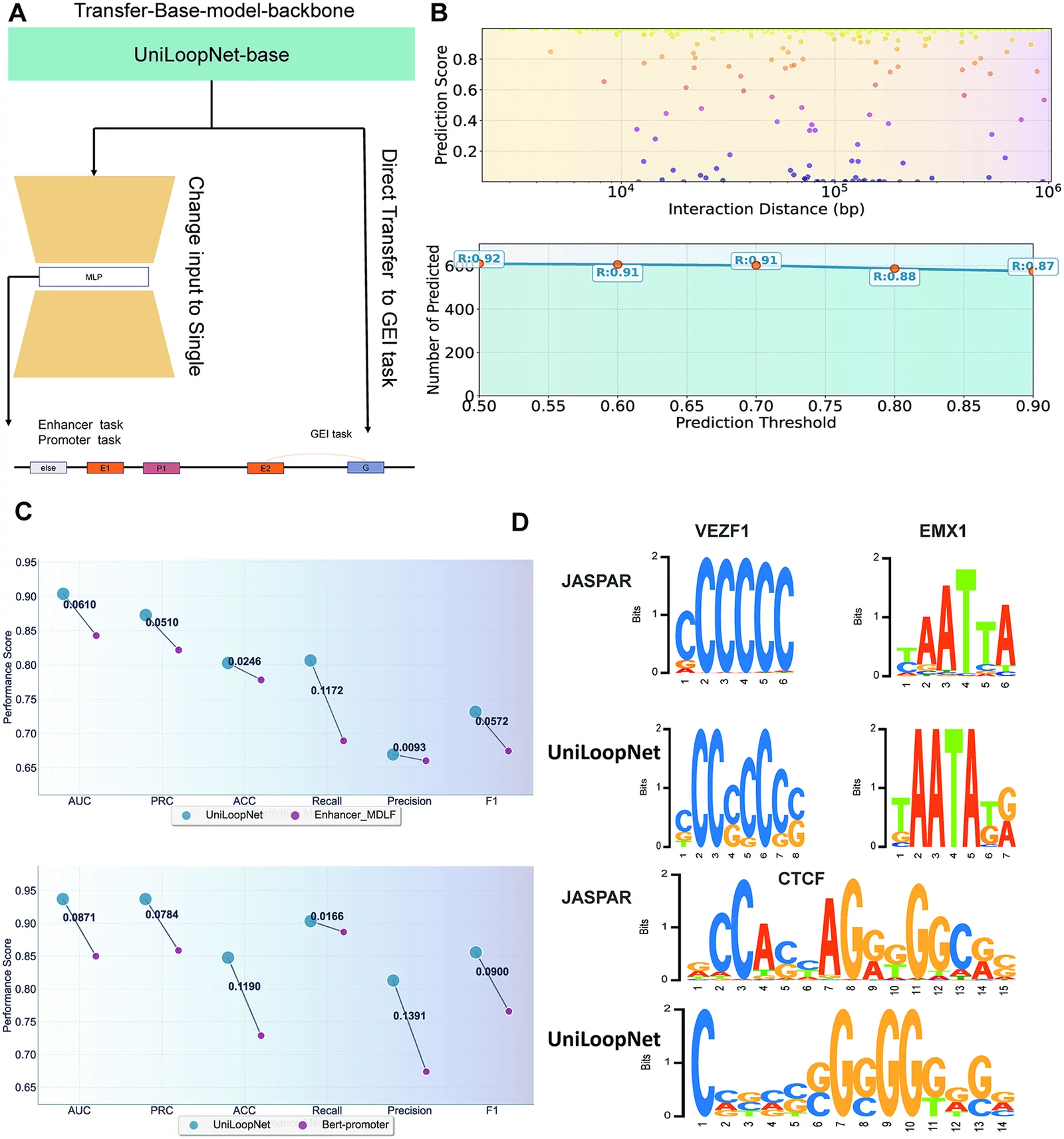
Transfer learning performance of UniLoopNet. **(A)** UniLoopNet-Transfer architecture implementing direct transfer for GEI prediction and backbone transfer for enhancer and promoter tasks. **(B)** Direct transfer prediction recall on 664 validated GEI positive samples, showing optimal performance at approximately 10 kb interaction distance, with stable recall across prediction thresholds (0.5–0.9). **(C)** Performance comparison for enhancer and promoter prediction tasks. UniLoopNet outperforms specialized methods (Enhancer-MDLF for enhancers, Bert-Promoter for promoters) across six evaluation metrics (AUROC, AUPRC, Accuracy, Recall, F1, Precision). **(D)** Comparison of UniLoopNet-learned motifs with JASPAR database entries, demonstrating biological validity of captured sequence features.

For enhancer and promoter prediction tasks, which exhibited high semantic similarity to the core EEI/EPI representations (cosine similarity 0.829 and 0.879), we implemented backbone transfer strategies that leverage pre-trained encoders while adapting to single-sequence inputs (Fig 4A). UniLoopNet consistently outperformed specialized methods across all evaluation metrics (Fig 4C): compared to Enhancer-MDLF, UniLoopNet achieved an AUC improvement of 0.061 for enhancer prediction, while surpassing Bert-Promoter with an AUC gain of 0.087 for promoter prediction. Both tasks showed similar improvements across precision, recall, and F1 scores, validating the effectiveness of backbone transfer for regulatory element prediction. In silico mutagenesis analysis revealed that positions contributing most significantly to model predictions coincided with known binding sites for VEZF1, EMX1, and CTCF transcription factors, showing strong concordance with JASPAR database motifs (Fig 4D). This alignment with established regulatory motifs confirms UniLoopNet’s biological interpretability and its capacity to capture functionally relevant sequence features through transfer learning approaches.

### UniLoopNet applies continual learning to divergent chromatin interaction types

To evaluate UniLoopNet’s extensibility and continual learning capability, we systematically assessed its performance on stripe-associated interaction prediction and CTCF-mediated interaction prediction, representing distinct chromatin spatial organization patterns. Stripe tasks are derived from continuous asymmetric contact patterns generated by unidirectional loop extrusion, while CTCF tasks focus on protein-mediated point-to-point interactions. We implemented incremental training with reduced epochs and learning rates while preserving pre-trained weights, continuously monitoring catastrophic forgetting of the original EPI/EEI tasks. To minimize forgetting and maintain prior task performance, we incorporated Elastic Weight Consolidation (EWC), which uses Fisher information matrices to identify and constrain important parameters from deviating from their optimal values for previous tasks (Fig 5A). CTCF-mediated interaction prediction yielded AUC of 0.9086 and AUPRC of 0.9256, while stripe prediction achieved AUC of 0.829 and AUPRC of 0.858 (Fig 5B).

**Fig 5:**
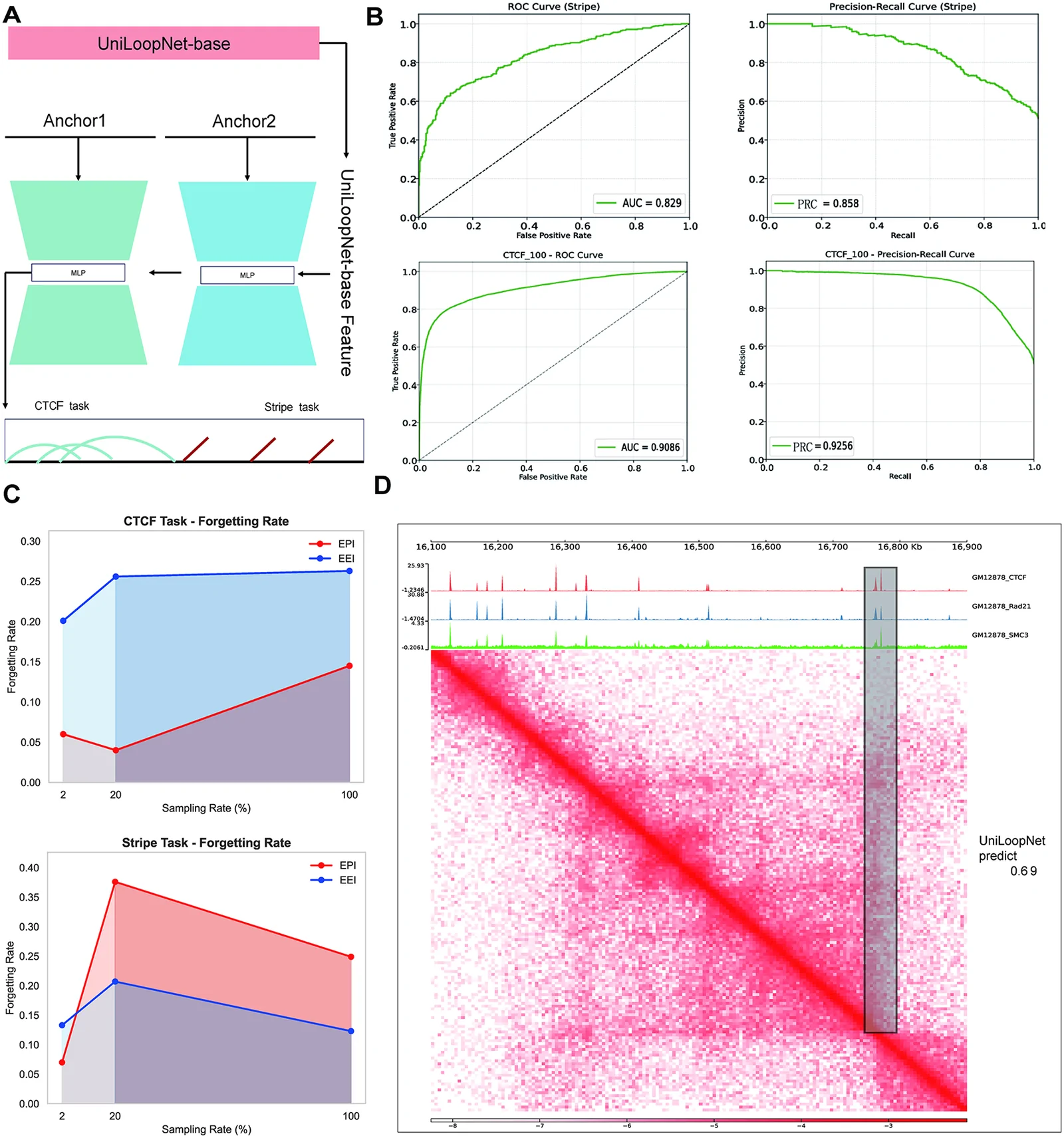
Continual learning performance of UniLoopNet. **(A)** UniLoopNet-Continual architecture for incremental task learning. **(B)** AUROC and AUPRC performance for stripe and CTCF-mediated interaction prediction. **(C)** Forgetting rates for original EEI and EPI tasks during continual learning of CTCF and stripe tasks. **(D)** UniLoopNet prediction for an experimentally validated promoter-enhancer stripe at the ATXN1 locus (chr6:16.1-16.9Mb). Hi-C contact map with corresponding CTCF, RAD21, and SMC3 ChIP-seq signals. The value shown in the panel (UniLoopNet predict = 0.69) is the model’s predicted probability that this locus harbors a stripe-associated interaction and is not a performance metric such as AUROC.

For CTCF tasks, average forgetting rates across 2%, 20%, and 100% sampling were 0.24 for EEI and 0.08 for EPI, with a combined average of 0.16. Stripe tasks showed forgetting rates of 0.15 for EEI and 0.23 for EPI, averaging 0.19 overall (Fig 5C). To validate biological relevance, we applied the stripe-trained model to previously identified promoter-enhancer stripes (PES), achieving a prediction probability of 0.69. Visualization using CoolBox revealed corresponding enrichment of CTCF, RAD21, and SMC3 signals at these stripe regions (Fig 5D, S2A Fig), consistent with cohesin-mediated loop extrusion mechanisms underlying stripe formation.

### UniLoopNet demonstrates few-shot learning capability across diverse genomic tasks

To systematically evaluate UniLoopNet’s few-shot learning performance, we assessed both transfer learning and continual learning strategies across four distinct tasks: enhancer prediction, promoter prediction, stripe-associated interaction prediction, and CTCF-mediated interaction prediction. We implemented task-specific adaptation strategies, employing backbone transfer for enhancer and promoter tasks given their high feature similarity with the core EEI/EPI representations, while utilizing continual learning for stripe and CTCF tasks given their comparatively lower similarity. All tasks were evaluated at 2%, 20%, and 100% sampling rates using multiple performance metrics including AUC, AUPRC, accuracy, and F1 score. For enhancer prediction, UniLoopNet substantially outperformed the specialized Enhancer-MDLF method across all sampling conditions, with the most dramatic improvements at 2% sampling: AUC increased by 0.202, AUPRC by 0.353, accuracy by 0.152, and F1 by 0.441 (Fig 6A). Similarly, for promoter prediction, UniLoopNet surpassed Bert-Promoter at all sampling rates, achieving remarkable gains at 2% sampling with AUC improvement of 0.359, AUPRC of 0.370, accuracy of 0.284, and F1 of 0.231 (Fig 6B).

**Fig 6:**
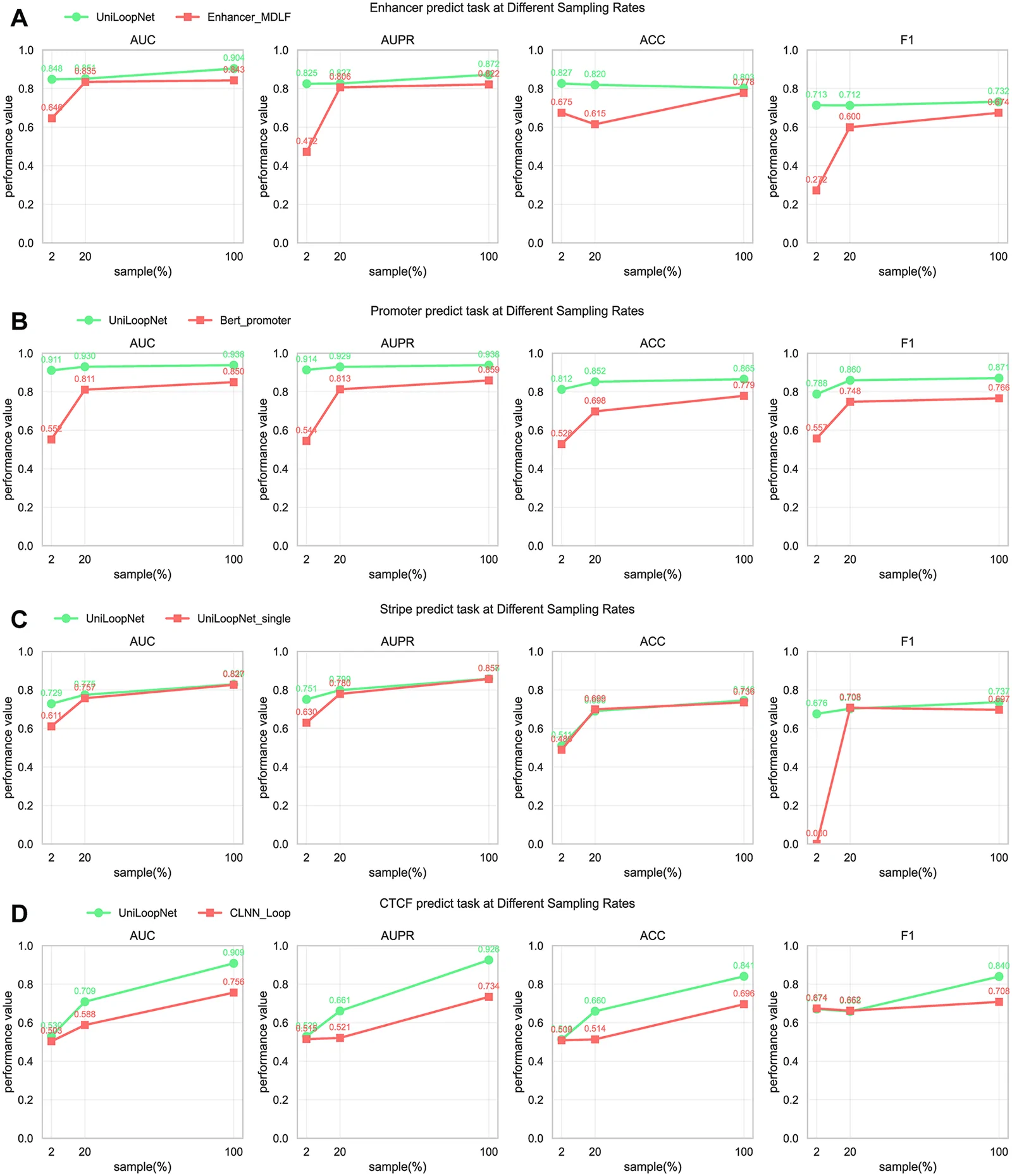
Few-shot learning performance of UniLoopNet. **(A)** Performance comparison between UniLoopNet-Transfer and Enhancer-MDLF for enhancer prediction at 2%, 20%, and 100% sampling rates across AUROC, AUPRC, Accuracy, and F1 metrics. **(B)** Performance comparison between UniLoopNet-Transfer and Bert-Promoter for promoter prediction at varying sampling rates. **(C)** Performance comparison between UniLoopNet-Continual and training from scratch for stripe prediction across sampling conditions. **(D)** Performance comparison between UniLoopNet-Continual and CLNN-Loop for CTCF-mediated interaction prediction at different sampling rates.

For stripe prediction, where no existing sequence-based methods are available for comparison, we benchmarked UniLoopNet’s continual learning approach against training from scratch. The continual learning strategy outperformed de novo training at 2% sampling: AUC improved by 0.118, AUPRC by 0.121, and F1 by 0.676 (Fig 6C). In CTCF-mediated interaction prediction, UniLoopNet exceeded the performance of CLNN-Loop; at 20% sampling, AUC increased by 0.121, AUPRC by 0.140, and accuracy by 0.146 (Fig 6D). These results demonstrate that both transfer and continual learning strategies enable effective knowledge reuse. The gains at low sampling rates (2% and 20%) highlight UniLoopNet’s data efficiency and its potential for practical applications where labeled genomic data is limited or expensive to obtain.

### Hyperparameter and architecture sensitivity analysis

To evaluate the robustness of UniLoopNet with respect to key hyperparameter selections and model architecture design, we performed a systematic sensitivity analysis focusing on the loss function hyperparameters and the CNN module architecture.

First, we investigated the effects of key hyperparameters in the loss function on model performance. The total loss function of UniLoopNet consists of classification loss, reconstruction loss, and continual learning regularization loss. The hyperparameter α controls the contribution of the reconstruction loss term Lrecon to the overall loss function, whereas λ regulates the constraint strength of the Elastic Weight Consolidation (EWC) regularization term LEWC during the continual learning stage. It should be noted that λ is introduced only during the continual learning stage and is therefore optimized exclusively for the CTCF and Stripe tasks, without affecting the training process of the basic EPI and EEI tasks.

To determine the optimal values of α and λ, we employed a grid search strategy to systematically evaluate different parameter combinations. The candidate values of α were set to {0.1, 0.3, 0.5, 0.7, 1.0}, while the candidate values of λ were set to {1000, 3000, 5000, 7000, 10000}. Since the magnitude of the EWC loss term is relatively small, a larger coefficient λ was adopted to ensure that its contribution remained at a comparable scale with other loss components. For each parameter combination, AUROC and AUPRC values were calculated on the corresponding test sets to evaluate model performance and stability. For the CTCF task, the model achieved the highest AUROC (0.9086) when α = 1.0 and λ = 5000. For the Stripe task, the same parameter combination achieved the best performance, with an AUROC of 0.8290 and an AUPRC of 0.8580. Considering the overall performance across both continual learning tasks, α = 1.0 and λ = 5000 were selected as the default hyperparameter configuration for all continual learning experiments in UniLoopNet (S3A–3D Fig).

Furthermore, to validate the rationality of the CNN module design, we performed a sensitivity analysis of the CNN architecture. Since the CNN module is responsible for extracting local regulatory patterns from DNA sequences, its convolutional depth, feature dimensions, and hidden representation space may influence the model’s ability to learn sequence features. Therefore, while keeping the input data, training strategy, optimization parameters, and other network components unchanged, we systematically evaluated the impact of different CNN configurations on UniLoopNet performance. Specifically, we tested different numbers of convolutional layers (2, 3, and 4 layers), different CNN channel scales (small, base, and large), and different hidden dimensions (256, 512, and 1024). All models were trained and evaluated using the same data partition strategy, and the mean AUC on the test set was used as the primary evaluation metric.

First, we analyzed the effect of CNN depth on model performance. Models with 2, 3, and 4 convolutional layers achieved mean AUC values of 0.9026, 0.9051, and 0.9021, respectively. Among them, the 3-layer CNN architecture achieved the best performance. Excessively deep architectures may introduce redundant feature learning or optimization difficulties due to increased model complexity, thereby providing no further performance improvement.

Next, we evaluated the influence of CNN channel scale on model performance. The results showed that the small-channel configuration substantially reduced model performance, achieving a mean AUC of 0.8794, suggesting that insufficient feature dimensions limit the model’s ability to represent complex regulatory sequence patterns. In contrast, increasing the channel scale to large achieved a mean AUC of 0.9049, which was comparable to the base configuration (0.9051). This indicates that increasing model capacity beyond the base configuration does not provide additional performance benefits while increasing computational costs. Additionally, we examined the effect of hidden dimension size. When the hidden dimension was set to 256, 512, and 1024, the corresponding mean AUC values were 0.8951, 0.9051, and 0.9029, respectively. These results indicate that a smaller hidden representation space limits the feature learning capability of the model, whereas further increasing the hidden dimension does not lead to additional performance gains. Therefore, a hidden dimension of 512 provides a better balance between representation capability and computational complexity. Based on these analyses, UniLoopNet ultimately adopted a 3-layer CNN architecture, the base channel configuration, and a hidden dimension of 512 as the default CNN design. This configuration achieves an optimal balance between predictive performance and model complexity (S3E Fig).

## Discussion

UniLoopNet advances the pair-classification paradigm for chromatin interaction prediction by establishing a multi-task framework that addresses multiple interaction types through a single shared architecture. Unlike previous pair-classification models that target individual interaction categories, UniLoopNet simultaneously handles enhancer-enhancer interactions, enhancer-promoter interactions, regulatory element identification, stripe-associated interactions, and CTCF-mediated interactions. By implementing task-specific adaptation strategies including multi-task learning, transfer learning, and continual learning, we demonstrated that UniLoopNet achieves superior performance across diverse interaction types. The framework’s data efficiency addresses a critical limitation in genomic modeling, enabling practical applications where labeled data is limited or expensive to obtain.

An important question is how pair-classification models such as UniLoopNet relate to sequence-to-map approaches, including Akita [24], DeepC [25], and Orca [26], that predict entire contact frequency matrices from continuous genomic sequences. These two paradigms address fundamentally different questions: sequence-to-map models capture the global contact landscape, distance-dependent decay, and the effects of large structural variants, whereas pair-classification models determine whether specific locus pairs interact and to which functional category an interaction belongs. The strengths of UniLoopNet lie in (i) task-specific classification with interpretable confidence scores, (ii) the ability to integrate knowledge across interaction categories through hierarchical transfer, and (iii) data efficiency. Conversely, UniLoopNet does not model intervening sequences or predict genome-wide contact matrices, which limits its ability to capture the mechanistic details of loop extrusion or the consequences of structural rearrangements. We view these paradigms as complementary: UniLoopNet’s interaction-type-resolved predictions could serve as high-confidence anchors to refine or validate the outputs of global contact prediction models.

Several limitations of the current framework merit discussion. First, as a sequence-only model, UniLoopNet captures regulatory potential encoded in DNA but cannot directly account for cell-type-specific epigenomic states. Chromatin interactions are dynamic and context-dependent; a sequence-based prediction reflects what is biochemically permissible rather than what is realized in a given cellular state. Retraining on cell-type-specific annotations is necessary for context-specific applications. Consistent with this, direct cross-cell-line transfer between GM12878 and K562 reduced performance, most markedly for EPI, whereas fine-tuning on target-cell-line labels restored performance to within-cell-line levels, indicating that the model requires cell-type-specific supervision to resolve cell-state-dependent interactions. Second, the anchor-based input strategy processes the two interacting loci independently without encoding the intervening sequence or the genomic distance between them. This design, shared by established pair-classification methods such as ChINN [18], TargetFinder [27], and SPEID [28], prioritizes scalability and cross-task generalizability, but it means that physical constraints—such as CTCF boundary insulation within the intervening region or distance-dependent contact decay—are not explicitly modeled. Our GEI analysis (Fig 4B) does reveal a distance-dependent performance profile, with optimal prediction near 10kb separation, suggesting that distance information is implicitly captured to some extent through anchor sequence composition. Third, our primary benchmarking employed balanced (1:1) positive-to-negative datasets following the protocols of TargetFinder, ChINN, and EnContact, which enables direct comparison with published methods. In additional evaluations, we complemented this design with distance-matched, anchor-swap, locus-disjoint, and realistically imbalanced (1:5) evaluation settings, under which the AUROC of UniLoopNet ranged from 0.8934 to 0.9366. In the 1:5 setting, AUPRC decreased to 0.7956 for EPI and 0.6935 for EEI, as expected with lower positive prevalence. Even a 1:5 ratio remains far denser than the genome-wide sparsity of true chromatin interactions, and all negative sets are computationally constructed rather than experimentally verified non-interactions; evaluation at genome-scale prevalence, together with explicitly GC- and composition-matched negative construction, therefore remains an important direction for future work. Fourth, the current framework does not encompass all levels of chromatin organization: topologically associating domains (TADs) and A/B compartments, which are typically defined by contact matrix features rather than locus-pair annotations, are not addressed here. Extending the framework to these higher-order structures is a natural direction for future development.

Our analysis of systemic lupus erythematosus GWAS variants demonstrates that UniLoopNet can resolve the effects of non-coding variants at the level of individual interaction types—a perspective that is not available from single-task models or from sequence-to-map predictions, which report aggregate contact changes without functional classification. For example, rs2069235 near the APOBEC3 cluster showed a consistent reduction in EEI scores across multiple target genes while exhibiting variable effects on EPI, suggesting that this variant may preferentially perturb enhancer-enhancer regulatory networks. We emphasize that these predictions generate testable hypotheses rather than experimentally validated conclusions; future work integrating allele-specific Hi-C or CRISPR perturbation data will be needed to confirm the interaction-type-specific effects predicted here.

The hierarchical adaptation strategy itself represents a reusable design pattern for multi-task genomic modeling. The quantitative semantic similarity analysis we developed (Fig 3D, 3E) provides a data-driven method for selecting the appropriate transfer strategy—direct prediction, backbone transfer, or continual learning—for any new task, based on its feature-space relationship to existing tasks. This removes the need for manual strategy selection and renders the framework extensible by design. Looking forward, our goal is to develop this into a broader foundation model that integrates anchor-based classification with distance-aware and epigenomic features. While current data augmentation approaches including complementary sequences, random shifts, and mutations can expand training sets, they often introduce redundant features that may not capture the full complexity of regulatory grammar. By leveraging UniLoopNet’s demonstrated shareability, scalability, and interpretability, we envision developing a framework that bridges the pair-classification and sequence-to-map paradigms, moving toward a more comprehensive approach for three-dimensional genome prediction across different biological contexts and research scenarios. Such integration promises to reveal novel insights into gene regulatory mechanisms and their roles in disease pathogenesis, advancing our understanding of developmental biology and precision medicine. Overall, UniLoopNet provides a robust and extensible framework for multi-type chromatin interaction prediction and interpretation, establishing a solid foundation for future research in gene regulatory mechanism analysis and disease-related molecular exploration.

## Supporting information

S1 Fig Performance comparison of UniLoopNet with baseline methods in GM12878 and K562 cell lines. Systematic comparison of classification performance between UniLoopNet and state-of-the-art multi-task methods (AlphaGenome, EPInformer, ChromBPNet) using five metrics (AUROC, AUPRC, accuracy, recall, and F1), together with comparisons between UniLoopNet and task-specific methods (EnContact for EEI, EPI-Trans for EPI) across the same metrics.
(TIF)

S2 Fig Stringent evaluation of UniLoopNet. (A) UniLoopNet predictions for experimentally validated promoter–enhancer stripes (PES): Hi-C contact maps at chr12:11.5–12 Mb (ETV6 locus) and chr11:116.6–117.2 Mb (SIK6 locus), each accompanied by CTCF, RAD21, and SMC3 ChIP-seq signals. (B) Performance of UniLoopNet under different evaluation strategies (chromosome-matched, distance-matched, realistic imbalanced, and anchor-swap test sets) for the EPI and EEI tasks. (C) Performance comparison of EPI and EEI prediction under cross-cell-line model transfer (K562 to GM12878, left; GM12878 to K562, right); for each category (EPI, EEI, and average AUROC), bars show direct prediction without fine-tuning, performance after fine-tuning for 15 epochs, and the reference model trained directly on the target cell line.
(TIF)

S3 Fig Hyperparameter and architecture sensitivity analysis. (A–B) AUROC and AUPRC of the CTCF task under different combinations of α and λ; (C–D) AUROC and AUPRC of the Stripe task under different α and λ combinations; the star marks the selected configuration (α = 1.0, λ = 5000). (E) Sensitivity analysis of the CNN architecture, including the number of convolutional layers, channel scales, and hidden dimensions.
(TIF)

S1 Table Details of UniLoopNet network architecture parameters. Complete layer-by-layer specification of the UniLoopNet architecture, including input/output tensor shapes and parameter counts for each module.
(PDF)

S2 Table Alleles at SLE risk loci exhibit distinct effects on EPI versus EEI. Prediction scores for each SNP before and after variant introduction, with delta values indicating the magnitude and direction of effect.
(PDF)

S3 Table Detailed information on UniLoopNet’s prediction of experimentally validated GEI. Prediction results for 664 functionally validated cis enhancer–gene pairs, together with recall at prediction thresholds from 0.5 to 0.9.
(PDF)

S4 Table Data sources. Summary of all datasets used in this study, including sample sizes, positive-to-negative ratios, and source repositories.
(PDF)

S5 Table Additional performance evaluations. Table A: comparison of model performance (AUROC and AUPRC) before and after enhancer–promoter locus-disjoint splitting. Table B: performance evaluation of UniLoopNet on the GEI dataset with matched negative controls. Table C: comparison between UniLoopNet and single-task models on the EPI and EEI prediction tasks.
(PDF)
