# Supplementary material for: An extensible deep learning framework for multi-type chromatin interaction prediction through hierarchical adaptation strategies
Source: PLoS Comput Biol. 2026 Oct 27;22(10):e1014911. doi: 10.1371/journal.pcbi.1014911 (PMC13730424; doi:10.1371/journal.pcbi.1014911)
Supplement: Supplementary file 1 [file pcbi.1014911.s004.pdf]

S1 Table

Details of UniLoopNet Network Architecture Parameters. Complete layer-by-layer specification of the UniLoopNet architecture, including input/output tensor shapes and parameter counts for each module.

Layer Type	Input Shape	Output Shape	Parameters
LayerType	InShape	OutShape	Params#
UniLoopNet	[1, 4, 5000]	[1, 4, 5000]	-
SequenceEncoder	[1, 4, 5000]	[1, 128, 151]	-
Sequential	[1, 4, 5000]	[1, 512, 1248]	-
Conv1d	[1, 4, 5000]	[1, 512, 4993]	16896
ReLU	[1, 512, 4993]	[1, 512, 4993]	-
MaxPool1d	[1, 512, 4993]	[1, 512, 1248]	-
Dropout	[1, 512, 1248]	[1, 512, 1248]	-
LayerNorm	[1, 1248, 512]	[1, 1248, 512]	1024
Sequential	[1, 512, 1248]	[1, 384, 310]	-
Conv1d	[1, 512, 1248]	[1, 384, 1241]	1573248
ReLU	[1, 384, 1241]	[1, 384, 1241]	-
MaxPool1d	[1, 384, 1241]	[1, 384, 310]	-
Dropout	[1, 384, 310]	[1, 384, 310]	-
Sequential	[1, 384, 310]	[1, 128, 151]	-
Conv1d	[1, 384, 310]	[1, 128, 303]	393344
ReLU	[1, 128, 303]	[1, 128, 303]	-
MaxPool1d	[1, 128, 303]	[1, 128, 151]	-
Dropout	[1, 128, 151]	[1, 128, 151]	-
SequenceEncoder	[1, 4, 5000]	[1, 128, 151]	-
Sequential	[1, 4, 5000]	[1, 512, 1248]	-
Conv1d	[1, 4, 5000]	[1, 512, 4993]	16896
ReLU	[1, 512, 4993]	[1, 512, 4993]	-
MaxPool1d	[1, 512, 4993]	[1, 512, 1248]	-
Dropout	[1, 512, 1248]	[1, 512, 1248]	-
LayerNorm	[1, 1248, 512]	[1, 1248, 512]	1024
Sequential	[1, 512, 1248]	[1, 384, 310]	-
Conv1d	[1, 512, 1248]	[1, 384, 1241]	1573248
ReLU	[1, 384, 1241]	[1, 384, 1241]	-
MaxPool1d	[1, 384, 1241]	[1, 384, 310]	-
Dropout	[1, 384, 310]	[1, 384, 310]	-
Sequential	[1, 384, 310]	[1, 128, 151]	-
Conv1d	[1, 384, 310]	[1, 128, 303]	393344
ReLU	[1, 128, 303]	[1, 128, 303]	-
MaxPool1d	[1, 128, 303]	[1, 128, 151]	-
Dropout	[1, 128, 151]	[1, 128, 151]	-
Conv1d	[1, 128, 151]	[1, 128, 151]	16512
Conv1d	[1, 128, 151]	[1, 128, 151]	16512
SequenceDecoder	[1, 128, 151]	[1, 4, 5000]	-
Sequential	[1, 128, 151]	[1, 200, 310]	-
Upsample	[1, 128, 151]	[1, 128, 302]	-
ConvTranspose1d	[1, 128, 302]	[1, 200, 310]	230600
ReLU	[1, 200, 310]	[1, 200, 310]	-
Sequential	[1, 200, 310]	[1, 200, 1248]	-
Upsample	[1, 200, 310]	[1, 200, 1240]	-
ConvTranspose1d	[1, 200, 1240]	[1, 200, 1248]	360200
ReLU	[1, 200, 1248]	[1, 200, 1248]	-

Layer Type	Input Shape	Output Shape	Parameters
Sequential	[1, 200, 1248]	[1, 4, 5000]	-
Upsample	[1, 200, 1248]	[1, 200, 4992]	-
ConvTranspose1d	[1, 200, 4992]	[1, 4, 5000]	7204
BatchNorm1d	[1, 4, 5000]	[1, 4, 5000]	8
Sigmoid	[1, 4, 5000]	[1, 4, 5000]	-
SequenceDecoder	[1, 128, 151]	[1, 4, 5000]	-
Sequential	[1, 128, 151]	[1, 200, 310]	-
Upsample	[1, 128, 151]	[1, 128, 302]	-
ConvTranspose1d	[1, 128, 302]	[1, 200, 310]	230600
ReLU	[1, 200, 310]	[1, 200, 310]	-
Sequential	[1, 200, 310]	[1, 200, 1248]	-
Upsample	[1, 200, 310]	[1, 200, 1240]	-
ConvTranspose1d	[1, 200, 1240]	[1, 200, 1248]	360200
ReLU	[1, 200, 1248]	[1, 200, 1248]	-
Sequential	[1, 200, 1248]	[1, 4, 5000]	-
Upsample	[1, 200, 1248]	[1, 200, 4992]	-
ConvTranspose1d	[1, 200, 4992]	[1, 4, 5000]	7204
BatchNorm1d	[1, 4, 5000]	[1, 4, 5000]	8
Sigmoid	[1, 4, 5000]	[1, 4, 5000]	-
DualTaskPredictor	[1, 128, 151]	[1, 1]	66048
AdaptiveAvgPool1d	[1, 128, 151]	[1, 128, 1]	-
AdaptiveAvgPool1d	[1, 128, 151]	[1, 128, 1]	-
Sequential	[1, 256]	[1, 256]	-
Linear	[1, 256]	[1, 512]	131584
ReLU	[1, 512]	[1, 512]	-
Dropout	[1, 512]	[1, 512]	-
Linear	[1, 512]	[1, 256]	131328
ReLU	[1, 256]	[1, 256]	-
Dropout	[1, 256]	[1, 256]	-
Sequential	[1, 256]	[1, 1]	-
Linear	[1, 256]	[1, 128]	32896
ReLU	[1, 128]	[1, 128]	-
Dropout	[1, 128]	[1, 128]	-
Linear	[1, 128]	[1, 1]	129
Sequential	[1, 256]	[1, 1]	-
Linear	[1, 256]	[1, 128]	32896
ReLU	[1, 128]	[1, 128]	-
Dropout	[1, 128]	[1, 128]	-
Linear	[1, 128]	[1, 1]	129
