# Supplementary material for: An extensible deep learning framework for multi-type chromatin interaction prediction through hierarchical adaptation strategies
Source: PLoS Comput Biol. 2026 Oct 27;22(10):e1014911. doi: 10.1371/journal.pcbi.1014911 (PMC13730424; doi:10.1371/journal.pcbi.1014911)
Supplement: Supplementary file 2 [file pcbi.1014911.s005.pdf]

S2 Table

Alleles at SLE risk loci exhibit distinct effects of risk variants on enhancer–promoter interactions (EPI) versus enhancer–enhancer interactions (EEI). Prediction scores for each SNP before and after variant introduction, with delta values indicating the magnitude and direction of effect.

SNP ID	Variant	Gene	Pre-EPI	Post-EPI	Δ(EPI)	Pre-EEI	Post-EEI	Δ(EEI)
rs3101018	C>T	VWA7	0.8246	0.8331	0.0086	0.9925	0.9924	-0.0001
rs3101018	C>T	MSH5	0.3043	0.3182	0.0139	0.9989	0.9989	0.0
rs9267544	C>A	C6orf48	0.0095	0.0095	0.0	0.9728	0.9706	-0.0026
rs9267544	C>A	HSPA1B	0.0001	0.0001	0.0	0.5238	0.5052	-0.0186
rs6643807	G>A	FAM50A	0.1917	0.1938	0.0022	0.0983	0.0945	-0.0039
rs2069235	G>A	APOBEC3A	0.8145	0.8042	-0.0103	0.1429	0.1258	-0.0171
rs2069235	G>A	APOBEC3B	0.5908	0.5756	-0.0152	0.9494	0.9429	-0.0064
rs2069235	G>A	APOBEC3C	0.1217	0.1152	-0.0065	0.9919	0.9911	-0.0008
rs2069235	G>A	APOBEC3D	0.4977	0.4825	-0.0153	0.9343	0.9261	-0.0083
rs2069235	G>A	ATF4	0.0095	0.0095	0.0	0.9728	0.9706	-0.0026
rs2069235	G>A	CBX6	0.0001	0.0001	0.0	0.82	0.798	-0.0219
rs2069235	G>A	CBX7	0.0408	0.0385	-0.0023	0.9766	0.9738	-0.0012
rs2069235	G>A	DNAL4	0.6848	0.6714	-0.0134	0.5048	0.4739	-0.031
rs2069235	G>A	FAM83F	0.0114	0.0108	-0.0006	0.9866	0.9847	-0.0019
