# Supplementary material for: An extensible deep learning framework for multi-type chromatin interaction prediction through hierarchical adaptation strategies
Source: PLoS Comput Biol. 2026 Oct 27;22(10):e1014911. doi: 10.1371/journal.pcbi.1014911 (PMC13730424; doi:10.1371/journal.pcbi.1014911)
Supplement: Supplementary file 3 [file pcbi.1014911.s006.pdf]

S3 Table

Detailed Information on UniLoopNet's Prediction of Experimentally Validated Gene-Enhancer Interactions (GEI). Prediction results for 664 functionally validated cis enhancer-gene pairs. The table presents the first 50 entries, while the complete dataset is available in the GEI_predictions file on GitHub.

Gene Chr	Gene Start	Gene End	Enh Chr	Enh Start	Enh End	#	Score	Positive	Distance
chr2	69688531	69901481	chr2	69056234	69056865	1	0.9907	Yes	738457
chrX	152990322	153010216	chrX	153250743	153251468	2	0.9999	Yes	250836
chr10	72432558	72522197	chr10	72426863	72427518	3	0.9950	Yes	50187
chr1	150521883	150533413	chr1	150517877	150518596	4	1.0000	Yes	9412
chr11	10326226	10328944	chr11	9573345	9573973	5	1.0000	Yes	753926
chr2	236402732	237040444	chr2	236369078	236369902	6	1.0000	Yes	352098
chr1	36273772	36323491	chr1	37209434	37209566	7	1.0000	Yes	910869
chr4	84457066	84527028	chr4	84551045	84551768	8	1.0000	Yes	59359
chr9	130628758	130640022	chr9	130624341	130625383	9	0.9999	Yes	9528
chr13	42846288	42897396	chr13	42833603	42834109	10	0.9980	Yes	37986
chr13	42846288	42897396	chr13	42832957	42833222	11	0.9999	Yes	38753
chr10	4934795	5025475	chr10	4983650	4984221	12	0.9999	Yes	3800
chr10	4934795	5025475	chr10	4908871	4909146	13	0.9995	Yes	71127
chr10	4934795	5025475	chr10	5062425	5062782	14	0.9995	Yes	82468
chr10	5029966	5060223	chr10	4983650	4984221	15	0.9997	Yes	61159
chrX	55035487	55057497	chrX	55143062	55143359	16	1.0000	Yes	96718
chr12	53817638	53825318	chr12	53815208	53815726	17	0.9996	Yes	6011
chr13	111530886	111567416	chr13	111609613	111610176	18	0.9980	Yes	60743
chr1	173578699	173639001	chr1	173640035	173640618	19	0.9998	Yes	31476
chr9	100745642	100778225	chr9	100723075	100723580	20	0.9994	Yes	38606
chr4	80822302	81046608	chr4	81048619	81048756	21	0.9734	Yes	114232
chr4	80822302	81046608	chr4	81064453	81064873	22	0.9504	Yes	130208
chr4	80822302	81046608	chr4	81100680	81101328	23	0.9839	Yes	166549
chr9	75766672	75785309	chr9	75758860	75759164	24	1.0000	Yes	16978
chr9	75766672	75785309	chr9	75764489	75764739	25	1.0000	Yes	11376
chr11	34874640	34938046	chr11	33966182	33966805	26	0.0019	No	939850
chr11	129939731	130014699	chr11	129876786	129877132	27	0.9986	Yes	100256
chr8	68085746	68255912	chr8	68278386	68278795	28	0.9999	Yes	107761
chr10	63661058	63856703	chr10	63539754	63540466	29	0.9998	Yes	218770
chr7	12726480	12730559	chr7	12762340	12762740	30	0.9998	Yes	34021
chr2	235401684	235405697	chr2	235421867	235422416	31	0.9999	Yes	18451
chr8	17913933	17942494	chr8	17980086	17980641	32	0.9980	Yes	52150
chr2	239335382	239360891	chr2	239329936	239330621	33	0.9999	Yes	17858
chr2	239335382	239360891	chr2	239330792	239331424	34	0.9992	Yes	17028
chr8	37962759	38001594	chr8	37948069	37948184	35	1.0000	Yes	34050
chr19	50431958	50437192	chr19	51106236	51106403	36	1.0000	Yes	671744
chr12	14518609	14651697	chr12	14410404	14411333	37	1.0000	Yes	174285
chr16	10420290	10577495	chr16	11325231	11325527	38	0.8947	Yes	826487
chr16	10420290	10577495	chr16	10591551	10591739	39	1.0000	Yes	92753
chr16	10420290	10577495	chr16	10416999	10417320	40	1.0000	Yes	81733
chr11	64662006	64684722	chr11	65185593	65186967	41	0.9897	Yes	512916
chr11	64662006	64684722	chr11	65188509	65189093	42	0.9921	Yes	515437
chr11	64662006	64684722	chr11	65247045	65247268	43	0.9990	Yes	573792
chr11	64662006	64684722	chr11	65239420	65239979	44	0.9999	Yes	566335
chr11	64662006	64684722	chr11	64240338	64241078	45	0.9998	Yes	432656
chr11	64662006	64684722	chr11	65261362	65261604	46	0.9992	Yes	588119

Gene Chr	Gene Start	Gene End	Enh Chr	Enh Start	Enh End	#	Score	Positive	Distance
chr3	182511287	182639423	chr3	182432966	182433055	47	0.9123	Yes	142345
chr3	182511287	182639423	chr3	182431923	182432188	48	0.9873	Yes	143300
chr3	141594965	141645356	chr3	141567842	141568701	49	0.9999	Yes	51889
chr3	141594965	141645356	chr3	141567550	141567694	50	0.9995	Yes	52538

Recall Performance at Different Thresholds

Model	Threshold	Count	Recall
UniLoopNet	0.5	(610/664)	0.9187
	0.6	(606/664)	0.9127
	0.7	(602/664)	0.9066
	0.8	(587/664)	0.8840
	0.9	(575/664)	0.8660
