# Supplementary material for: An extensible deep learning framework for multi-type chromatin interaction prediction through hierarchical adaptation strategies
Source: PLoS Comput Biol. 2026 Oct 27;22(10):e1014911. doi: 10.1371/journal.pcbi.1014911 (PMC13730424; doi:10.1371/journal.pcbi.1014911)
Supplement: Supplementary file 4 [file pcbi.1014911.s007.pdf]

S4 Table

Data Sources. Summary of all datasets used in this study, including sample sizes, positive-to-negative ratios, and source repositories. The sample sizes and positive-to-negative ratios reported in the table are based on the original datasets before floor-based balancing and therefore do not reflect the sample counts after balanced sampling.

GM12878 Cell Line

Type	Sample Size	Pos:Neg Ratio	Source
EEI	6,311	1:1	FANTOM5
EPI	3,801	1:1	TargetFinder
Stripe	8,851	1:1	Expresso
CTCF	184,088	1:1	ChINN
Enhancer	7,917	1:1	Enhancer-MDLF
Promoter	6,764	1:1	Bert-Promoter
GEI	664	Positive only*	Gasperini et al.

** The GEI dataset contains experimentally validated positive samples; a matched negative control set of non-interacting enhancer–gene pairs was additionally constructed for the revised evaluation (Table B in S5 Table).*

K562 Cell Line

Type	Sample Size	Pos:Neg Ratio	Source
EEI	15,670	1:1	FANTOM5
EPI	3,952	1:1	TargetFinder

ChIP-seq Data

Type	Format	Source
CTCF	bigwig	Roadmap Epigenomics
RAD21	bigwig	Roadmap Epigenomics
SMC3	bigwig	Roadmap Epigenomics
