# Supplementary material for: An extensible deep learning framework for multi-type chromatin interaction prediction through hierarchical adaptation strategies
Source: PLoS Comput Biol. 2026 Oct 27;22(10):e1014911. doi: 10.1371/journal.pcbi.1014911 (PMC13730424; doi:10.1371/journal.pcbi.1014911)
Supplement: Supplementary file 5 [file pcbi.1014911.s008.pdf]

S5 Table

Table A. Comparison of model performance (AUROC and AUPRC) before and after enhancer–promoter locus-disjoint splitting.

	EEI Test	EPI Test	EEI-AUROC	EEI-AUPRC	EPI-AUROC	EPI-AUPRC
Original	701	422	0.904	0.8984	0.9061	0.9062
Enhancer–promoter locus-disjoint	623	370	0.8934	0.8767	0.8967	0.8872

Table B. Performance evaluation of UniLoopNet on the GEI dataset with matched negative controls.

	AUROC	AUPRC	Recall	Specificity	FPR
GEI	0.8922	0.8957	0.8182	0.8182	0.1818

Table C. Comparison between UniLoopNet and single-task models on the EPI and EEI prediction tasks.

	AUROC	AUPRC
UniLoopNet-EPI	0.9061	0.9062
UniLoopNet-EEI	0.904	0.8984
Single-EPI	0.8898	0.8981
Single-EEI	0.8911	0.8854
